# Commissioning and quality assurance for the treatment delivery components of the AccuBoost system

**DOI:** 10.1120/jacmp.v16i2.5156

**Published:** 2015-03-08

**Authors:** Ileana Iftimia, Mike Talmadge, Ron Ladd, Per Halvorsen

**Affiliations:** ^1^ Radiation Oncology Department Lahey Hospital and Medical Center Burlington MA; ^2^ Tufts University School of Medicine Boston MA USA

**Keywords:** AccuBoost system, treatment delivery components, commissioning methodology, quality assurance tests

## Abstract

The objective for this work was to develop a commissioning methodology for the treatment delivery components of the AccuBoost system, as well as to establish a routine quality assurance program and appropriate guidance for clinical use based on the commissioning results. Various tests were developed: 1) assessment of the accuracy of the displayed separation value; 2) validation of the dwell positions within each applicator; 3) assessment of the accuracy and precision of the applicator localization system; 4) assessment of the combined dose profile of two opposed applicators to confirm that they are coaxial; 5) measurement of the absolute dose delivered with each applicator to confirm acceptable agreement with dose based on Monte Carlo modeling; 6) measurements of the skin‐to‐center dose ratio using optically stimulated luminescence dosimeters; and 7) assessment of the mammopad cushion's effect on the center dose. We found that the difference between the measured and the actual paddle separation is <0.1 cm for the separation range of 3 cm to 7.5 cm. Radiochromic film measurements demonstrated that the number of dwell positions inside the applicators agree with the values from the vendor, for each applicator type and size. The shift needed for a good applicator‐grid alignment was within 0.2 cm. The dry‐run test using film demonstrated that the shift of the dosimetric center is within 0.15 cm. Dose measurements in water converted to polystyrene agreed within 5.0% with the Monte Carlo data in polystyrene for the same applicator type, size, and depth. A solid water‐to‐water (phantom) factor was obtained for each applicator, and all future annual quality assurance tests will be performed in solid water using an average value of 1.07 for the solid water‐to‐water factor. The skin‐to‐center dose ratio measurements support the Monte Carlo‐based values within 5.0% agreement. For the treatment separation range of 4 cm to 8 cm, the change in center dose would be <1.0% for all applicators when using a compressed pad of 0.2 cm to 0.3 cm. The tests performed ensured that all treatment components of the AccuBoost system are functional and that a treatment plan can be delivered with acceptable accuracy. Based on the commissioning results, a quality assurance manual and guidance documents for clinical use were developed.

PACS numbers: 87.55.Qr, 87.56.Da, 87.90.+y

## I. INTRODUCTION

The AccuBoost system (Advanced Radiation Therapy, Tyngsboro, MA) is comprised of a mammography unit that has been modified to accommodate a set of tungsten alloy surface applicators designed for use with a high dose rate (HDR) ${\;}^{192}\text{Ir}$ remote afterloader. Circular (standard and skin dose‐optimized (SDO)) and D‐shaped applicators of various sizes are designed with a channel running along the interior periphery so as to collimate the HDR ${\;}^{192}\text{Ir}$ source's photons and to optimize the dose distribution at depth. The system is intended to deliver noninvasive targeted brachytherapy to the lumpectomy cavity either as an alternative to an electron boost^1^, ^2^ employed during external beam treatment, or as a monotherapy form of partial breast irradiation.^(3)^ In both cases, the breast is first immobilized using moderate compression prior to each treatment. A radiographic image is then acquired in order to localize the lumpectomy cavity, as well as to select both the type and position of the applicators to be used. With the breast properly immobilized by the opposing mammography compression paddles, a pair of applicators is then positioned on either side of the breast, facing inward. A 2D indexing mechanism is used to position the applicators based on the location of the surgical cavity.

The thickness of the compressed breast is determined by the separation distance as measured on the mammography unit and this, along with the type and size of the applicator and the source activity, is used to plan the treatment. The system is designed for a separation range of 3 cm to 10 cm (with a recommended maximum value of 8 cm for APBI due to skin dose tolerance). Treatment planning is accomplished using a nomogram based on Monte Carlo (MC) modeling developed by Rivard et al. (M.J. Rivard, personal correspondence via email, April 2013), which determines dwell times and position to deliver a given dose at the midplane of the compressed breast. While confirmatory measurements were performed by Yang and Rivard^(4)^ and Rivard et al.,^(5)^ we are not aware of any published or independent recommendations for clinical commissioning of the AccuBoost system. We therefore conducted an extensive set of commissioning measurements in our clinic to validate the performance for the treatment delivery components of the AccuBoost system prior to clinical use. A quality assurance (QA) program was then developed based on the findings from our commissioning process. This paper describes our commissioning process, results, and the QA program.

The commissioning process included: a) acceptance testing of the system upon completion of manufacturer's installation; b) full physics evaluation of the mammography and CR components by a mammography‐qualified medical physicist; c) assessment of the accuracy of the separation value displayed by the mammography system (which is used to compute the HDR ${\;}^{192}\text{Ir}$ source dwell times to deliver the prescribed dose); d) validation of the number of dwell positions within each applicator using GafChromic‐type radiochromic film (Ashland, Wayne, NJ); e) assessment of the accuracy and precision of the applicator localization system; f) assessment of the combined dose profile of two opposed applicators in the treatment position to ensure that they are coaxial and that the center of the radiation profile agrees with the location of the applicators as indicated on the localization calipers; g) determination of a method for absolute dose measurement from the HDR ${\;}^{192}\text{Ir}$ source; h) measurement of the absolute dose delivered with each AccuBoost applicator to confirm acceptable agreement with the manufacturer's stated dose based on MC modeling; i) skin‐to‐center (midplane) dose measurements using optically stimulated luminescence dosimeters (OSLDs) (Landauer Inc., Glenwood, IL); and j) mammopad (Hologic Inc., Bedford, MA) cushion effect on the center dose.

Acceptance testing and routine QA tests for the imaging components of the AccuBoost system follow the standard methodology for a mammography unit in magnification stand mode, and will not be reviewed here.

## II. MATERIALS AND METHODS

Prior to clinical use, commissioning measurements were taken to validate the performance of the treatment delivery system. Various tests were developed, as described below, to ensure that all treatments components of the AccuBoost system are functional and can be relied upon to deliver treatments accurately.

### A. Separation check

The vendor calibration of the unit was performed with a breast phantom advanced only about half way onto the magnification stand to mimic the usual breast position between the paddles. Our measurements were performed in a similar manner, per vendor recommendations. Using acrylic slabs, various separations were created from ∼3 cm to 8 cm. The slab pile was placed on the AccuBoost mammography unit and compressed until a readout for the compression force was displayed on the unit. The separation value displayed on the unit at that compression force was recorded and compared with the known thickness of the acrylic phantom.

Using Rivard's nomograms for standard and SDO round and D–shaped applicators, it was estimated that a 0.1 cm variation in separation (for the usual separation range of 4 cm to 6 cm) could result in up to 2.0% change in total dwell time and corresponding dosimetric effect. Based on this observation, it was decided that the test results would be acceptable if the separation error will be less than or equal to 0.1 cm for the usual separation range mentioned above.

### B. Applicators validation

All applicators were visually inspected for signs of damage. The proper internal catheters were trimmed and inserted inside each applicator where they were secured using a locking screw. The total length of the source guide tube plus internal catheter was verified to be 130 cm, using the HDR manufacturer's calibrated length wire.

Validation of the dwell positions within each applicator was performed using radiochromic film. Strips of films were cut with a width of ∼1 cm and a length equal to that of the internal circumference of each applicator. Plans were generated in BrachyVision 11 (Varian Medical Systems, Palo Alto, CA) for each applicator size, using a dwell time of 3 to 4 s per position, and exported to the HDR unit (Gamma Med iX, Varian Medical Systems) using channels 1 and 2. A pair of applicators was placed on a flat surface in the HDR ${\;}^{192}\text{Ir}$ treatment room, with the internal catheters facing up. Films were placed on‐edge along the internal circumference of each applicator and secured in place using tape. After exposure, the films were removed and the number of dwell positions was counted and compared with the vendor data for each applicator type and size. Given that it was somewhat difficult to resolve the adjacent dwell positions, new films were generated exposing only the dwell positions #1,3,…,n, for “n” odd number, and #1,3,…,n−1, n, for “n” even number, where “n” was total number of dwell positions for a given applicator. The dwell time per position was increased to 5 s.

### C. Applicator‐grid alignment check

Applicator‐grid alignment was verified for both round and D‐shaped applicators by using vendor‐provided overlay templates corresponding to each applicator that were taped on the underside of the mammography unit's paddle‐mounted localization grid. Both applicators (on the “paddle side” and “magnification stand side”) were mounted and aligned with the template using indexing two‐dimensional applicator positioning mechanisms that were adjusted to achieve the best possible alignment with the respective template. Any measureable positional deviation between the applicators and the template was then visually estimated. Based on the findings from these tests, a recommendation was made to the physicians regarding a minimum margin around the target.

### D. Dry‐run test using film

The purpose of this test was to assess the combined dose profile of two opposed applicators in the treatment position to ensure that the dose distributions are coincident and correctly centered in the transverse plane. A $10\times 10\;{\text{cm}}^{2}$ piece of radiochromic film was cut and placed on the AccuBoost grid and centered on the reference location. The center of the film was marked and a pair of applicators was mounted into the mammography paddle. A plan was generated using two applicators and appropriate number of dwell positions for that applicator size, with 12 s per dwell position. The procedure was performed for a selection of round (standard and SDO) and D‐shaped applicators. After the exposure was performed, the film was scanned in transmission mode with a resolution of 300 dpi using an Epson Perfection V700 Photo dual lens system (Epson America, Long Beach, CA) and analyzed with the ImageJ software (ImageJ, National Institutes of Health, Bethesda, MD) to determine if there was any measurable shift of the dosimetric center relative to that used by the image guidance and applicator positioning systems. The analysis was performed by taking two orthogonal profiles and measuring the shift between the profile dosimetric center and the geometric center marked on the film. The shift was converted from pixels to millimeters. In addition, the width of the high‐dose region of the profile was compared to the internal diameter of the applicator.

### E. Determination of an absolute dose measurement method

Our institution does not possess a custom‐manufactured phantom for measuring dose from the HDR ${\;}^{192}\text{Ir}$ source. With standard phantoms (rectangular slabs of polystyrene and solid water, and rectangular water tanks), the most appropriate ionization chamber in our inventory is the PTW 23343 Markus parallel‐plate chamber (PTW Freiburg GmbH, Freiburg, Germany). Baltas et al.^(6)^ indicate that a slab phantom can be used for absolute dose measurements with acceptable accuracy, though a phantom scatter correction factor may be necessary. In their 2009 article describing experimental validation of the MC modeling of D‐shaped AccuBoost applicators, Yang and Rivard^(4)^ used a model 23343 Markus chamber for their absolute dose measurements using an N_D,W_ ADCL factor for ${\;}^{60}\text{Co}$, with acceptable results. This was further corroborated by Rivard et al.^(5)^ for the round applicators. A discussion with the authors confirmed that they used a ${\;}^{60}\text{Co}\;N_{D,W}$ factor without attempting to establish a $k_{Q}$ factor for ${\;}^{192}\text{Ir}$, and they did not employ a phantom correction factor for the solid phantoms used. They measured dose in polystyrene and compared these results to MC simulations also in polystyrene to validate the MC simulations in breast tissue for clinical dose calculations.

Tedgren and Carlsson^(7)^ investigated different phantom materials for ${\;}^{192}\text{Ir}$ dosimetry and found a significant dependence upon phantom dimensions, recommending that a phantom correction factor be experimentally determined for the specific phantom type and dimensions used at each institution.

Based on this recommendation, we obtained an $N_{D,W}$ calibration factor for ${\;}^{60}\text{Co}$ for our Markus chamber and performed an initial set of measurements in a Solid Water phantom with the HDR ${\;}^{192}\text{Ir}$ source in a 5F catheter placed in a solid water slab with a groove fitting the catheter. The dose measurements for single‐dwell irradiations confirmed the appropriateness of this methodology for determining the absolute dose at a known distance from the HDR ${\;}^{192}\text{Ir}$ source.

#### E.1 Measurements in water

**Figure 1:**
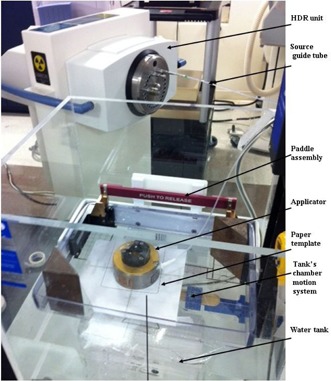
Setup for the dose measurements in water. A small 1D motorized water tank was used for this purpose (see text for details).

Since the phantom dimensions and characteristics can have a significant effect on these measurements, we decided to first perform measurements in water, at 1.5 cm and 4 cm depths, for one applicator of each dimension. A small 1D motorized water tank (Standard Imaging, Middleton, WI) was used for this purpose and placed on our Acuity conventional simulator couch (Varian Medical Systems), which resides within the HDR ${\;}^{192}\text{Ir}$ brachytherapy treatment room (see setup photo in Fig. 1). A 0.1 cm thick waterproof buildup cap was screwed over the Markus chamber. The water tank was filled so as to provide about 15 cm depth of water for backscatter, as suggested in the joint AAPM‐ESTRO TG‐229 report.^(8)^ The chamber was affixed to a custom holder mounted to the tank's one‐dimensional motion system and then centered at a given depth in water using the simulator's lasers and graticule. Two blocks of acrylic were placed in the water to support the AccuBoost mammography paddle, which was placed above the chamber but just below the surface of the water to ensure that no air gaps would be present. Templates were created on paper for each applicator size using copies of the vendor‐provided templates. The central part of these templates was removed and the template was centered on the paddle (using the simulator's lasers and graticule), the applicator then being placed over the paper template. The chamber was manipulated using the tank's chamber motion system until it touched the paddle. This position was set as the “origin”, and then the chamber was moved downward to the desired depth, taking into account the inherent 0.1 cm water proof buildup cap (which was approximated here as being water equivalent).

Plans were generated in BrachyVision 11 using a single applicator connected to channel 1. The number of dwell positions and the dwell time values were taken from the Rivard's nomogram for polystyrene at the same geometric depth and applicator size. The dwell times were set to give a dose of 1.00 Gy in polystyrene, for 100% normalization. The Markus chamber was *a priori* calibrated for ${\;}^{60}\text{Co}$ in water. The calibration factor for ${\;}^{192}\text{Ir}$ is 1.0% to 2.0% lower than the calibration factor for ${\;}^{60}\text{Co}$,^(1)^ but this effect was neglected. $P_{\text{pol}}$ and $P_{\text{ion}}$ were *a priori* measured in solid water and polystyrene at different depths, and both were found to be within 0.4% of unity. Consequently, both $P_{\text{pol}}$ and $P_{\text{ion}}$ were assumed to be equal to 1.000 for these dose measurements in water. Corrections were made for electrometer, temperature, and pressure. The measured dose in water was converted to dose in polystyrene, and then compared with the nomogram data:

(1) $$\text{Dose to polystyrene}=\text{Rdg}.\;\text{in water}\times N_{D,w}^{{\text{Co}}_{60}}\times P_{\text{pol}}\times P_{\text{ion}}\times P_{\text{el}}\times P_{T,P}\times {\left(\frac{\bar{\mu_{en}}}{\rho }\right)}_{w}^{poly}.$$

Based on our prior experience with HDR ${\;}^{192}\text{Ir}$ dosimetry and published literature,^9^, ^10^ it was decided that the agreement between our dose measurements and vendor data should be within ±6.0% to deem the results acceptable.

#### E.2 Measurements in solid water

The measurements in water were quite elaborate and time consuming. With the intent to generate baseline values for our annual quality assurance (AQA) dose tests, measurements were also performed in solid water for each applicator, at 1.5 cm and 4 cm depths (corresponding to a treatment separation of 3 cm and 8 cm, respectively). A Markus chamber in a 2.5 cm polystyrene slab was used for measurements, with a 5 cm solid water slab for backscatter. A 0.3 cm slab of polystyrene was placed on the top of the phantom to mimic the 0.367 cm paddle (by direct measurements it was found that the 0.3 cm polystyrene slab is ~ equivalent to the paddle thickness). The plans generated in BrachyVision 11 (as described in Materials & Methods section E 1 above) were also used for these tests. For the Markus chamber, a calibration factor for ${\;}^{60}\text{Co}$ in water was used (a calibration factor in solid water could not be obtained), neglecting the fact that for ${\;}^{192}\text{Ir}$ the factor may be lower, as previously discussed. $P_{\text{pol}}$ and $P_{\text{ion}}$ were assumed to be equal to 1.000 for these dose measurements. Corrections were made to account for the electrometer, temperature, and pressure.

**Figure 2:**
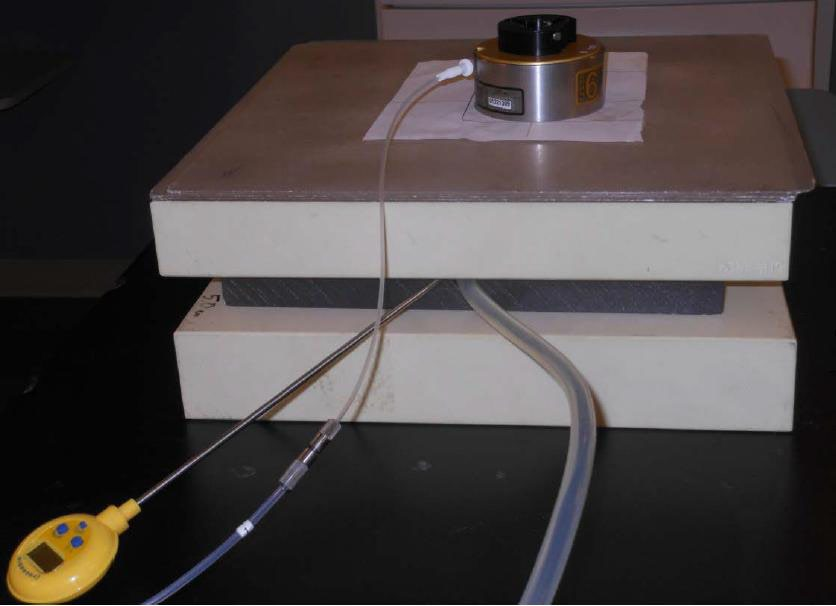
Setup for the dose measurements in solid water used to obtain the phantom factor. A paper template was centered on the top of the phantom over the ion chamber using the Acuity lasers and graticule, and then the applicator was placed over the template.

Measurements were performed on the Acuity conventional simulator couch. The lasers and graticule were used to center the chamber. For these measurements, paper templates were centered on the top of the phantom over the ion chamber using the lasers and graticule to achieve alignment, and then the applicator was placed over the template and connected to the HDR ${\;}^{192}\text{Ir}$ unit (see Fig. 2).

The ratio of the measurements in water and solid water was used to obtain a solid water‐to‐water factor (i.e., phantom factor), which takes into account the dimensions and shape of the Solid Water phantom. Also, it takes into consideration the fact that the calibration factor from the ADCL and the mass energy absorption coefficient are for water, respectively polystyrene to water, not for solid water/polystyrene to solid water. The presence of the 2.5 cm polystyrene slab used for the chamber (which may have a different contribution than the solid water to the backscatter) and any depth setup errors are also included in this factor. This phantom factor will be used for future AQA tests to convert the measurements in solid water to dose in polystyrene:

(2) $$\text{Dose to polystyrene}=\text{Rdg}.\;\text{in SW}\times N_{D,w}^{{\text{Co}}_{60}}\times P_{\text{pol}}\times P_{\text{ion}}\times P_{\text{el}}\times P_{T,P}\times {\left(\frac{\bar{\mu_{en}}}{\rho }\right)}_{w}^{poly}\times \text{phantom factor}.$$

The mass energy absorption coefficient ${\left(\frac{\bar{\mu_{en}}}{\rho }\right)}_{water}^{poly}=0.97$ in Eqs. (1) and (2) above.^(4)^

### F. Skin‐to‐center (midplane) dose measurements using OSLDs

OSLDs were used to experimentally verify the skin‐to‐center dose (SCD) ratios provided by the manufacturer. Unscreened OSLDs were screened in‐house using a 1.00 Gy exposure to 6 MV X‐rays to determine sensitivity adjustment factors for each dosimeter. The OSLD‐reader system was then calibrated using 6 MV X‐rays produced by a linear accelerator calibrated in accordance with the AAPM TG‐51 protocol.^(11)^ Testing for 6 and 15 MV X‐rays, as well as 6, 9, 12, 16, and 20 MeV electron energies, showed an uncertainty of ∼2.0% (k=1). OSLDs are reused after being annealed using a fluorescent light to less than 0.01 Gy with a useful life limited to a cumulative dose of less than 10.00 Gy. Initial testing with an HDR ${\;}^{192}\text{Ir}$ source indicated absolute dose accuracy within ∼10% at distances greater than 1 cm. The uncertainty in determining the relative SCD ratios with the OSLDs was, therefore, judged to be acceptably low.

For each measurement, a midplane dose of 2.00 Gy was delivered to the 90% isodose line using two parallel‐opposed, equally weighted beams. The dwell times for each applicator were obtained from the nomogram provided by Rivard. The dose and percentage normalization were selected based on that used clinically for breast boost treatments. Measurements were performed using phantoms comprised of flexible tissue‐equivalent bolus material, providing some compressibility. For each measurement, two OSLDs were placed along the central axis of the two applicators, one at the phantom midplane and the other at the phantom surface. Measurements were undertaken for the smallest and largest of each applicator type (standard round or D‐shaped) and for phantom thicknesses approximating 3 cm, 8 cm, and 10 cm separation.

**Figure 3:**
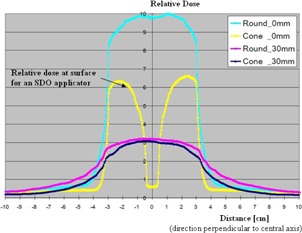
Dose profiles at two different depths for an SDO versus a standard round applicator obtained by Rivard et al. (personal correspondence via email, April 10, 2014)^)^ using Monte Carlo modeling (printed with permission). The SDO dose profile at the surface (shown in yellow) was used as a guide for our placement of the OSLDs in an attempt to measure maximum surface dose.

Spot check measurements were also performed for the SDO round applicators to confirm the vendor‐provided SCD ratios. The midplane dose measurement was performed by placing an OSLD along the central axis; however, the surface measurements were made by placing two OSLDs approximately 0.8 cm to 1.0 cm radially inward of the applicator's inner circumference in an attempt to measure maximum surface dose based on the dose distributions provided by the vendor (see Fig. 3).

**Figure 4:**
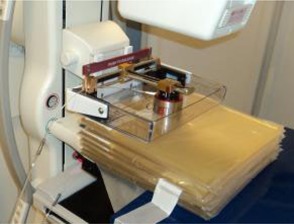
Setup for the skin‐to‐center dose measurements using OSLDs and a phantom comprised of flexible tissue‐equivalent bolus material. For each measurement, two OSLDs were placed along the central axis of the two applicators, one at the phantom midplane and the other at the phantom surface.

Since AccuBoost treatments typically utilize two equally‐weighted beam pairs, the alternate beam pair can be expected to contribute dose to the off‐axis skin areas as well, though to a much lesser extent. While this contribution was expected to be relatively insignificant from a clinical perspective, a set of basic measurements was performed to validate this hypothesis. These measurements were made by placing OSLDs on the lateral aspect of bolus phantom previously described, placed in such a way so as to simulate the clinical setup when orthogonal beam pairs are used and provide a measurement of the dosimetric contribution to a maximally exposed skin region from treatment by an off‐axis beam pair. Two measurements were performed using the standard 6 cm round applicator at separations of 4.3 cm and 7.9 cm, as well as a third measurement using the standard 7 cm round applicator at 8.0 cm of separation. The setup used for the OSLD measurements is shown in Fig. 4.

### G. Mammopad cushion effect on the center dose

Mammopad foam cushions provided by the vendor are used to increase patient comfort and breast stability during treatment. The separation displayed on the mammography unit includes the mammopad thickness. Since the separation is an essential parameter used for AccuBoost treatment planning, an evaluation of the mammopad cushion's dosimetric effect was undertaken.

The CT number for the pad was found to be (either compressed or uncompressed) very close to air (∼−900 HU). The thickness of the uncompressed pad is ∼0.6 cm. The thickness of the pad becomes ∼0.2 cm to 0.3 cm when compressed in a clinical setting. Measurements were performed for various applicators using solid water (at 1.5 cm and 4 cm depth, corresponding to a treatment separation of 3 cm and 8 cm, respectively). A Markus chamber in a 2.5 cm polystyrene slab was used, with a 5 cm solid water slab for backscatter. A slab of solid water (1.5 cm or 4 cm thick) was placed over the ion chamber. The applicator was carefully centered relative to the Markus chamber and connected to the HDR ${\;}^{192}\text{Ir}$ unit. Using Rivard's nomogram, a single‐channel plan was generated for the corresponding applicator type and size, and dwell times corresponding to a separation equal to 2*depth. The reading was used to obtain the center dose ($D_{c}$). The measurement was repeated by adding a 0.6 cm solid water, and then by replacing the 0.6 cm solid water with an uncompressed mammopad, for the same dwell time corresponding to a separation equal to $2^{*}\text{depth}+0.6\;(\text{cm})$. A dimensionless pad factor was obtained, which takes into consideration the increase in dose caused by the lack of attenuation in the air‐like cushion material. This factor, along with data measured by adding a 0.2 cm or a 0.3 cm solid water to the standard depth and dwell times corresponding to $2^{*}\text{depth}+0.2\;(0.3)\;(\text{cm})$, were used to extrapolate the previous results to the situation when a compressed mammopad (i.e., thickness ∼0.2 cm to 0.3 cm) is used. The center dose in the presence of mammopad $\left(D_{c}^{\prime }\right)$ was obtained and compared to the center dose ($D_{c}$) when no mammopad was used. Details regarding the measurements and theoretical analysis are provided in the Appendix A.

## III. RESULTS & DISCUSSION

### A. Separation check

Measurements showed a difference between the measured and the actual paddle separation <0.1 cm for the separation range of 3 cm to (~) 7.5 cm, and <0.15 cm for a separation >7.5 cm. Based on our established tolerance, test results were considered to be acceptable.

### B. Applicator validation

**Figure 5:**
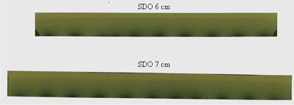
Examples of dwell position check films for an SDO 6 cm applicator (upper panel) and for an SDO 7 cm applicator (lower panel).

All applicators were in good condition without any sign of damage. The radiochromic films demonstrated that all the source dwell positions were within the internal circumference of the applicators as intended, for each applicator type and size. Figure 5 shows examples of dwell position check films for applicators with even and odd number of dwell positions (SDO round 6 cm and 7 cm, respectively). Per the vendor's recommendation, the step size for the AccuBoost treatment plans is 1 cm. The number of dwell positions within the internal circumference of the SDO 6 cm and 7 cm applicator should be 18 and 21, respectively. As seen on the films displayed in Fig. 5, all exposed positions can be counted [i.e., 10 positions (#1,3,…17, and 18) for the SDO 6 cm applicator, and 11 dwell positions (#1,3,…19, and 21) for the SDO 7 cm applicator].

### C. Applicator‐grid alignment check

The observed deviation in applicator‐grid alignment was within 0.2 cm (including setup/reading errors, visual estimation, and the potential imperfection of the plastic templates). Based on this uncertainty, a clinical recommendation was made that treatment plans should use of a minimum margin of 0.5 cm around the target.

### D. Dry‐run test using film

**Figure 6:**
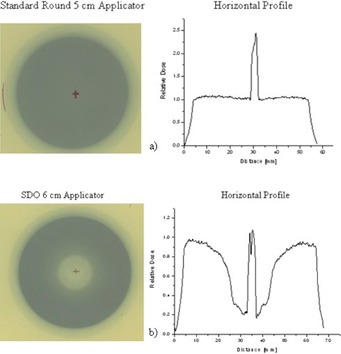
Examples of dry‐run tests of treatment delivery accuracy and applicator coaxial alignment for (a) a standard round 5 cm applicator, and (b) an SDO round 6 cm applicator. The profiles were used to determine the shift between the dosimetric and the geometric center of the applicator.

The dry‐run test using film was performed for a selection of round (standard and SDO) and D‐shaped applicators. Figure 6 shows examples of dry‐run tests of treatment delivery accuracy and applicator alignment for a) a standard round, and b) an SDO round applicator. The corresponding horizontal profiles are also displayed. The mark on the film, added during the experimental setup, generated a distortion on the image (a much larger pixel value comparative to surrounding points), which was used to assess the shift. With this method, we found that the shift of the dosimetric center is within 0.15 cm, and the width of the high‐dose region of the profiles correlated well with the internal diameter of each applicator.

### E. Dose measurements

Dose measurements in water converted to polystyrene, as shown in Eq. (1), agreed within 5.0% (see Table 1) with Rivard's data in polystyrene for the same applicator type, size, and treatment depth. The measurements were performed at 1.5 cm and 4 cm depth, corresponding to the two extremes of the separation used clinically (3 cm and 8 cm, respectively).

A solid water‐to‐water factor (phantom factor) was obtained for each applicator by comparing the measurements in solid water and water. Based on the fact that these values were within about ±1.2% for all round and D‐shaped applicators for the clinical separation range of 3 cm to 8 cm (see Table 2), it was decided to use an average factor of 1.07 for all future AQA measurements. For these tests, the dose measurements will be performed in solid water and converted to polystyrene, as shown in Eq. (2) above. The measured dose was rounded to two significant figures in 1, 2.

**Table 1:** Measured dose in water converted to polystyrene vs. Monte Carlo dose values in polystyrene

*Applicator*	*Depth (cm)*	*Meas. Dose in Water (Gy)*	*Meas. Dose Conv. to Polystyrene (Gy)*	*Monte Carlo Dose in Polystyrene (Gy)*	*% Diff.*
Std Round	1.5	0.99	0.96	1.00	−4
5 cm	4.0	0.99	0.96	1.00	−4
Std Round	1.5	0.98	0.95	1.00	−5
6 cm	4.0	0.98	0.95	1.00	−5
Std Round	1.5	1.00	0.97	1.00	−3
7 cm	4.0	1.00	0.97	1.00	−3
D‐shaped	1.5	0.99	0.96	1.00	−4
45 mm	4.0	0.99	0.96	1.00	−4
D‐shaped	1.5	1.00	0.97	1.00	−3
53 mm					

**Table 2:** Solid water‐to‐water (phantom) factors

*Applicator*	*Depth (cm)*	*Meas. Dose in Solid Water Conv. To Polystyrene(Gy)*	*Meas. Dose in Water Conv. to Polystyrene (Gy)*	*Phantom Factors* ^a^
Std Round	1.5	0.90	0.96	1.07
5 cm	4.0	0.89	0.96	1.08
Std Round	1.5	0.90	0.95	1.06
6 cm	4.0	0.90	0.95	1.07
Std Round	1.5	0.91	0.97	1.07
7 cm	4.0	0.90	0.97	1.08
D‐shaped	1.5	0.90	0.96	1.07
45 mm	4.0	0.90	0.96	1.06
D‐shaped	1.5	0.92	0.97	1.05
53 mm	4.0	0.92	0.97	1.06

^a^ Average phantom factor 1.066±0.013 (approximated as 1.07)

The variation in the measured dose for applicators of the same type and size was within 0.5%. Based on this observation, it was decided that the AQA tests should be performed for only one applicator with a given dimension and type (standard round, SDO round, or D‐shaped).

The commissioning tests were initially performed for the standard round 5, 6, and 7 cm diameters, and for D‐shaped 45 and 53 mm applicators provided. At a later date, the standard round 8 cm, D‐shaped 60 mm, and the SDO round applicators (size 5 cm, 6 cm, 7 cm, and 8 cm) were provided. The commissioning for those applicators followed the same procedures as described in Materials & Materials section above, but the dose measurements were performed in solid water only, using the phantom factor previously obtained to convert the dose to polystyrene.

### F. OSLD measurements

The OSLD measurements were used to determine SCD ratios and compare to those predicted by the MC model (results for the standard round and D‐shaped applicators can be found in Table 3, while those from the SDO applicators are listed in Table 4). The measurements were performed for the smallest and largest applicators, for a few separations in the clinical range of 3 cm to 8 cm, and also for a 10 cm separation. Rivard's nomogram can be used for a separation up to 10 cm. In our clinic it was decided not to treat patients with a separation more than 8 cm because of the increase in skin dose. The agreement between the SCD measured values and MC‐based data was within 5.0% for all applicators and separations used for this test.

The experimental setup used resulted in separation distances of 0.2 cm to 0.4 cm in excess of those listed in 3, 4. In order to correct for this, linear interpolation or extrapolation, when necessary, of the MC data was performed to estimate the expected SCD ratio for the experimental separation distances. The result of the off‐axis skin dose measurements showed that the contribution from the orthogonal beam pair was estimated to be approximately 5% to 8% of the skin dose received from the on‐axis beam pair.

Considering the treatment as a whole (two equally weighted orthogonal beam pairs) and assuming a perfectly repeatable setup between fractions, as well as no overlap between the fields, the SCD values must be divided by a factor of two to estimate the skin dose to the four hypothetical skin areas that would receive maximum dose. Additionally, these values can then be scaled by approximately 5% to 8% in order to account for the off‐axis beam pair contribution to skin dose.

**Table 3:** OSLD measurements: skin‐to‐center ratio (standard round and D‐shaped applicators)

*Separation (cm)/Applicator*	*Round 5 cm*	*Round 8 cm*	*D 45 mm*	*D 60 mm*
3.0	SCD Measured	1.22	1.08	1.15	1.12
	SCD MC	1.26	1.13	1.21	1.16
	% Diff	−3.4%	−4.8%	−4.6%	−3.6%
8.0	SCD Measured	2.57	1.85	2.21	1.88
	SCD MC	2.59	1.87	2.30	1.96
	% Diff	−0.7%	−1.1%	−3.8%	−4.0%
10.0	SCD Measured	3.45	2.25	2.98	2.45
	SCD MC	3.35	2.32	3.01	2.47
	% Diff	3.1%	−3.0%	−1.1%	−0.8%

**Table 4:** OSLD measurements: skin‐to‐center ratio (SDO round applicators)

*Applicator*	*SDO Round 5 cm*	*SDO Round 8 cm*
Separation	8.0 cm	5.0 cm
SCD Measured	2.01	1.21
SCD MC	2.06	1.25
% Diff	−2.6%	−3.4%

The results from the OSLD measurements support the MC‐based SCD values that are provided by the vendor for the standard round, D‐shaped, and SDO round applicators and also elaborate on these by providing an estimate of the contribution to the skin dose from the off‐axis beam pair. Overall these results underscore the importance of considering the SCD values, as well as the overall skin dose, when planning an AccuBoost treatment.

### G. Mammopad cushion effect on the center dose

Table 5 shows the percentage change of the center dose for a standard 6 cm round applicator (see Appendix A for the notations in the table). As expected, the percentage change decreases if the depth increases or/and the thickness of the compressed mammopad decreases. Tests performed for other applicators showed that the applicator used did not appreciably change the results.

The measurements showed that in the treatment separation range of 4 cm to 8 cm, the change in center dose would be <1.0% for all applicators when using a compressed pad of 0.2 cm to 0.3 cm. Based on these results, it was decided to use a separation as displayed on the unit (which includes the mammopad thickness) for the treatment planning when using the mammopad for the patients.

**Table 5:** Percentage change of the center dose caused by the Mammopad for a standard 6 cm round applicator (see Appendix A for notations)

*Depth (cm)*	$R_{1}[nC]$	$R_{1}^{\prime }[nC]$	$R_{3}^{\prime }[nC]$	$D_{c}∼2R_{1}$	$D_{c}^{\prime }∼R_{1}^{\prime }+R_{3}^{\prime }$	*% Change*
1.5 cm+0.3 cm pad	2.032	2.147	1.986	4.06	4.13	1.7
4.0 cm+0.3 cm pad	2.005	2.090	1.957	4.01	4.05	0.9
1.5 cm+0.2 cm pad	2.032	2.098	1.979	4.07	4.08	0.3
4.0 cm+0.2 cm pad	2.005	2.062	1.964	4.01	4.03	0.4

### H. QA manual

A QA manual for the treatment delivery components of the AccuBoost system was developed based on the commissioning results. This manual describes the routine QA tests to be performed, the minimum frequencies for each test, the individual responsible for each test, and the acceptable limits for each test (see Table 6). Because the applicator internal catheters are more sensitive than other components of the AccuBoost system, it was decided to test the applicators monthly, quarterly, and annually. The separation readout and dose constancy will only be checked annually. The dose will be measured in solid water for one applicator of each size and type and one separation, and then converted to dose in polystyrene. The tolerance for the AQA dose constancy was set to ±7.0%, taking into consideration the uncertainty for the phantom factor.

In the event that any QA test produces a result that is outside of the acceptable limits, the test should be repeated. Reproducible results that are beyond acceptable limits require corrective action, as described in the procedure for each test. During one year of routine QA testing following this program, we have found the tests to be a reasonable balance of process efficiency and predictive power. For example, the paddle separation test identified a minor leveling issue for the upper paddle, which was brought to the attention of the vendor's field service engineer for adjustment.

The experience gained during commissioning also proved valuable in developing appropriate process checklists and applicator selection guidelines. For example, we found that the applicator position can shift slightly during the mechanical process of locking the applicator into the holder on each paddle, so we have incorporated a final visual check of applicator centering after mounting, using a handheld mirror and flashlight. Similarly, the information gained from commissioning led to concise guidelines for applicator selection, including a method to evaluate the depth of the surgical cavity from the skin surface in each projection based on the other projection's localization image (particularly relevant for the SDO applicator type).

**Table 6:** Annual quality assurance program for the treatment delivery components of the AccuBoost system

*QA Test (Performed by Authorized Medical Physicist)*	*Minimum Frequency*	*Acceptable Results*
Visual inspection of applicators	Monthly	Functional, no kinks
Pathway length for each applicator plus Source Guide Tube	Quarterly	≤0.2 cm from nominal
Catheter condition / replacement	Annually or after any replacement	If replaced, trim length and verify dwell positions with film
Dose constancy (one separation per applicator)	Annually	±7.0% of baseline values (Monte Carlo data from manufacturer)
Separation readout accuracy	Annually	≤0.2 cm from nominal

## IV. CONCLUSIONS

The tests performed ensured that all treatment components of the AccuBoost system are functional and that a treatment plan can be delivered with acceptable accuracy, with an overall spatial accuracy of 0.2 cm and measured doses agreeing with MC calculations within 5.0%. Based on the commissioning results, a quality assurance manual and guidance documents for clinical use were developed, and have proven effective based on one year of routine use.

## Appendix A: Mammopad cushion effect on the center dose

a) Measurements were performed using a Markus chamber in a Solid Water phantom at 1.5 cm and 4.0 cm depths, as described in the Materials & Methods section G. A plan was generated with the dwell times corresponding to a separation equal to 2*depth. The center dose $D_{c}$ is given by:
  (A1) $$D_{c}\approx 2\times R_{1},$$
  where $R_{1}$ is the ion chamber reading.
b) The measurement was repeated by adding a 0.6 cm solid water, using dwell times corresponding to a separation equal to 2*depth +0.6 (cm).
c) The measurement described in item b) above was repeated by replacing the 0.6 cm solid water with an uncompressed mammopad, for the same dwell times.
  A dimensionless pad factor was obtained, as shown in Eq. (A2):
  (A2) $$R_{3}=R_{2}\times (1+\Delta )\Rightarrow \Delta =\frac{R_{3}}{R_{2}}-1$$
  where $R_{2}$ and $R_{3}$ are the ion chamber reading from items b) and c) above, respectively.
  A theoretical assessment was performed to extrapolate the results for a compressed mammopad (i.e., thickness ∼0.2 cm to 0.3 cm). It was concluded that for such small pad thicknesses the term Δ varies approximately linearly with the pad thickness (i.e., the pad factor becomes Δ/n, where n equals 2 and 3 for a 0.3 cm and a 0.2 cm compressed pad, respectively).
d) The measurement was then repeated by adding a 0.2 cm or a 0.3 cm solid water to the standard depth of 1.5 cm or 4 cm, using dwell times corresponding to 2*depth +0.2 (0.3) (cm).
  The ion chamber reading measured in solid water (R_2_) was converted to a value (R^′^_3_) as if the 0.2 (0.3) cm thickness of solid water is replaced with a compressed pad.
  (A3) $$R_{3}^{'}=R_{2}\times (1+\Delta /n),$$
  where *n* equals 2 and 3 for a 0.3 cm and a 0.2 cm compressed pad, respectively. The center dose % change is given by:
  (A4) $$\%\text{change}=\frac{\left(D^{'}{\;}_{c}-D_{c}\right)}{D_{c}}(\%)=\frac{R^{'}{\;}_{1}+R^{'}{\;}_{3}}{2\times R_{1}}-1$$
  (A5) $$\text{where}\;R^{'}{\;}_{1}=R_{1}\times \frac{\text{Total Dwell Time}(\text{for a separation of}\;2\times \text{depth}+\text{pad})}{\text{Total Dwell Time}(\text{for a separation of}\;2\times \text{depth})}.$$
